# Unlocking the Antiadipogenic Potential of Carotenoids From *Galdieria phlegrea*

**DOI:** 10.1002/biof.70027

**Published:** 2025-06-05

**Authors:** Enrica Giustino, Paola Imbimbo, Jenifer Trepiana, Maria Puy Portillo, Daria Maria Monti

**Affiliations:** ^1^ Department of Chemical Sciences University of Naples Federico II Naples Italy; ^2^ Nutrition and Obesity Group, Department of Nutrition and Food Science, Faculty of Pharmacy and Lucio Lascaray Research Center University of the Basque Country (UPV/EHU) Vitoria‐Gasteiz Spain; ^3^ CIBERobn Physiopathology of Obesity and Nutrition Institute of Health Carlos III Madrid Spain; ^4^ BIOARABA Health Research Institute Vitoria‐Gasteiz Spain

**Keywords:** 3T3‐L1, adipogenesis, biorefinery, carotenoids, cosmetics, *Galdieria phlegrea*

## Abstract

Oxidative stress, a sedentary and inactive lifestyle, or a high‐fat diet are among the main causes responsible for the alteration of skin topography, a complex multifactorial disorder of the deeper layers of the skin, mainly caused by an impairment of lipid production, degradation, and storage. Natural bioactive compounds can improve skin health and appearance as well as reassure consumers' about the safety of the molecules used in cosmetics. Carotenoids from microalgae are receiving attention because of their potent antioxidant activity. Here, a carotenoid extract from *Galdieria phlegrea* residual biomass was successfully explored for its ability to inhibit in vitro skin hyperpigmentation, to interfere with 3T3‐L1 adipocytes differentiation, as well as to affect de novo lipogenesis and enhance lipolysis in mature adipocytes. The same set of experiments was done in parallel on the extract obtained from fresh biomass, but no biological activity was observed. The obtainment of a high‐value class of molecules in a cascade approach highlights the importance of a biorefinery approach to obtain active extracts with a concurrent lowering of the overall process costs.

## Graphical abstract

The carotenoid extract from *Galdieria phlegrea* residual biomass inhibits 3T3‐L1 adipocyte differentiation, affects the de novo lipogenesis, and enhances lipolysis in mature adipocytes.

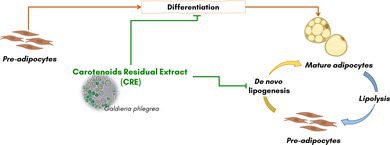

## 1 Introduction

The alteration of the skin topography, also known as cellulite or gynoid lipodystrophy (GLD), is a complex and sophisticated multifactorial disorder of the deeper layers of the skin [1]. Despite it has always been considered a harmless skin condition that only affects its appearance and esthetics, it is now conceived as a real clinical condition, due to a strict link to oxidative stress, the involvement of microcirculation failure, and inflammatory processes [2]. The pathology is mainly caused by an impairment of lipid production, degradation, and storage in response to external stimuli, such as a sedentary and inactive lifestyle or a high‐fat diet [3, 4, 5]. An increase in adipocytes size and their proliferation can dramatically change the cell and tissue morphology, thus resulting in the alteration of the skin structure, as well as in the oxidation and inflammation of the entire tissue [6]. Therefore, a relevant contribution to the amelioration of cellulite could be achieved by using molecules that can either faster the lipolysis in mature adipocytes or slow down the pre‐adipocyte differentiation [2].

Furthermore, skin exposure to UV radiation can negatively affect the skin's appearance, leading to the breakdown of elastin and collagen, increasing skin hyperpigmentation and causing premature skin aging [7, 8]. Over the last years, the research moved forward the use of natural bioactive compounds in the cosmeceuticals to improve skin health and appearance along with consumers' concerns about chemically synthesized molecules in cosmetics [9]. Indeed, the use of nonbiodegradable organic solvents, normally involved in chemical synthesis, besides being not eco‐friendly, may also lead to contamination of the final product, thus pushing consumers' preferences toward natural products [10]. Plants and seaweeds represent a natural reservoir of phytomolecules that are responsible for plant color, relevant for plant physiology as they are involved in biotic and abiotic oxidative stress defense [11, 12]. Among these, a prominent role is played by flavonoid glycosides and anthocyanins, typically produced by higher plants as a defense mechanism against stressors [11] or polyphenols, polysaccharides, and secondary metabolites (e.g., alkaloids, terpenoids) produced by seaweeds [13]. From a biotechnological point of view, this class of molecules acts as antioxidants, which may be used as antiaging, sun protecting factors, or anticellulite ingredients in cosmetic formulations, as they counteract ROS‐induced pathologies by different mechanisms of action [14]. However, plant‐based products present several issues, mainly related to cultivation (in terms of space required and time) and extraction procedures, which are time consuming and economically expensive [9, 15]. In this scenario, the use of bioactive molecules extracted from microalgae could be a perfect alternative. It is well established that microalgae are a reliable source of a wide variety of environmentally friendly and safe bioactive molecules (i.e., phycobiliproteins, carotenoids, PUFAs, polysaccharides, and sulphated polysaccharides) endowed with different and peculiar biological activities [16, 17, 18, 19]. Microalgae are also able to live in different environments and under different harsh conditions, and they do not compete with arable lands [20]. In photosynthetic organisms, carotenoids are responsible for light harvesting and energy quenching, and they may represent up to 14% of algal biomass [21]; during the last years, carotenoids received increasing attention because of their potent antioxidant activity and potential function in preventing adverse health conditions in humans, thus finding applications in cosmeceuticals [22]. The global carotenoid market is projected to reach USD 2.33 billion by 2032, exhibiting a Compound Annual Growth Rate (CAGR) of 3.76% (https://www.fortunebusinessinsights.com/industry‐reports/carotenoids‐market‐100180, March 20, 2025), in particular, for astaxanthin, β‐carotene, zeaxanthin, lutein, and fucoxanthin [23]. Topical or oral use of astaxanthin can suppress skin hyperpigmentation and improve the condition of all skin layers [24], whereas fucoxanthin and neoxanthin are able to interfere with adipocyte differentiation [19]. Even if microalgae can be easily grown, and their metabolism can be modulated by changing external parameters (no genetic manipulation is required), their use at an industrial scale is still a challenge because of the costs related to upstream and downstream processes [25, 26]. The obtainment of high‐value products in a cascade approach could pave the way for their industrial exploitation. In our previous work, a complete and effective green biorefinery, starting from the thermo‐acidophilic red microalga *Galdieria phlegrea*, was set up. The strategy was designed to extract bioactive molecules starting from the one with the highest market value (i.e., Phycocyanin) and decreasing the polarity of the solvent in each step to obtain a carotenoid extract and a lipid fraction [27]. The obtained carotenoid extract, mainly composed of zeaxanthin and β‐carotene, was proven to be endowed with strong antioxidant activity on a cell‐based system [27]. In the present paper, we focused our attention on carotenoids extracted from *G. phlegrea* residual biomass after protein extraction. The strength of using a whole extract, without any purification step, could represent a further advantage with respect to chemically synthesized molecules, in which purification is mandatory [28]. Indeed, the reduction of unit operations would result in a reduction in time and costs.

For this reason, a carotenoid extract from *G. phlegrea* residual biomass was explored for its in vitro antioxidant and antityrosinase activity, as well as to study its effect in counteracting adipocyte differentiation and affecting lipid metabolism in mature adipocytes.

## 2 Materials and Methods

### 2.1 Reagents

All reagents, unless differently specified, were purchased from Sigma‐Aldrich (St. Louis, MO, USA).

### 2.2 Carotenoids Extraction

*G. phlegrea* (strain 009) was provided from the Algal Collection of the University Federico II (ACUF, http://www.acuf.net). Carotenoid extraction was performed on dry biomass, either on the fresh biomass and on the biomass after protein extraction (residual biomass), as previously reported by Imbimbo et al. [27]. Briefly, 200 mg of freeze‐dried biomass were resuspended in 5 mL of pure ethanol and disrupted by ultrasound‐assisted extraction for 4 min on ice (40% of the amplitude instrument, Bandelin Sonopuls HD 3200). After sonication, the extraction volume was adjusted to 20 mL and macerated overnight at 4°C in the dark. Carotenoids were then recovered in the supernatant by centrifugation at 12,000*g* for 10 min. Extracts were dried under nitrogen stream and weighed to obtain the extraction yields, expressed as the percentage of the ratio between g of extract and g of dried biomass. Total carotenoid content was evaluated spectrophotometrically according to Gilbet‐Lopez [29] and expressed as the percentage of the ratio between mg of carotenoids and mg of extract.

### 2.3 In Vitro Antioxidant ABTS Assay

To evaluate carotenoids antioxidant activity, an in vitro spectrophotometric assay was performed according to Re [30]. Carotenoid extracts, tested at different concentrations, were allowed to react with ABTS^**•**+^ for 7 min in the dark, and the absorbance was measured at 734 nm. Trolox (6‐hydroxy‐2,5,7,8‐ tetramethylchromane‐2‐carboxylic acid) was used as a standard to obtain a calibration curve. The results obtained are expressed as IC_50_ value, that is, the concentration of the extract able to inhibit 50% of the radical.

### 2.4 In Vitro Antityrosinase Assay

The in vitro antityrosinase activity was evaluated as described by Imbimbo et al. [31] and carotenoids extracted from fresh and residual *G. phlegrea* biomass were tested. Kojic acid was used as the standard inhibitor. Values are reported as IC_50_ values (mg/mL), that is, the concentration of extract able to inhibit 50% of tyrosinase activity.

### 2.5 Cell Culture and Biocompatibility Assay

Murine preadipocytes (3T3‐L1) were purchased from ATCC (Manassas, VA, USA) and cultured in Dulbecco's modified Eagle's medium (DMEM) (GIBCO, BRL Life Technologies, Grand Island, NY), supplemented with 10% fetal bovine serum, 2 mM l‐glutamine, and antibiotics, at 37°C in a 5% CO_2_ humidified atmosphere. The biocompatibility of carotenoids extracted both from fresh and residual biomass was evaluated by the MTT assay. 3T3‐L1 pre‐adipocytes were seeded in a 96‐well plate at a density of 3 × 10^3^ cell/well and incubated with increasing concentrations of each extract (0.5–100 μg/mL) for 24 and 48 h. At the end of incubation, cell viability was assessed by the MTT assay, as previously reported [32]. Cell viability was expressed as the percentage of viable cells in the presence of the extract compared to the controls, represented by untreated cells and cells incubated with identical volumes of DMSO.

### 2.6 Adipocyte Differentiation

To efficiently differentiate preadipocytes into their adipocyte phenotype, 3T3‐L1 cells were seeded in six‐well plates at a density of 1.5 × 10^5^ cells/well and allowed to reach confluence. Two days after confluence (Day 0), differentiation was induced by adding DMEM supplemented with 0.5 mM 3‐isobutyl‐1‐methylxanthine (IBMX), 10 μg/mL insulin, and 1 μM dexamethasone. After 2 days, cells were incubated with DMEM supplemented with 1 μg/mL insulin. From Day 4 to the end of differentiation, cells were grown in DMEM supplemented with 0.2 μg/mL insulin, freshly replaced every 48 h [33]. To analyze the effect of carotenoid extract on differentiation, cells were coincubated with 25, 50, or 75 μg/mL of carotenoids for the whole time of the experiment (from Days 0 to 8). At the end of the experiment, cells were extensively washed with 0.01 M PBS pH 7.4, and triglyceride (TG) accumulation, gene, and protein expression were analyzed. Images of cells during differentiation were acquired by an Olympus CH optical microscope (Olympus, Tokyo, Japan) at ×10 magnification.

### 2.7 Effect of Carotenoids on Mature Adipocytes

To test the effects of carotenoid extract on mature adipocytes, cells were differentiated as previously reported until Day 12 and then treated with 25, 50, or 75 μg/mL of carotenoid extract for 24 h. At the end of incubation, cells were extensively washed with 0.01 M PBS pH 7.4, and TG accumulation, gene, and protein expression were analyzed. Images of cells at the end of differentiation were acquired by an Olympus CH optical microscope (Olympus, Tokyo, Japan) at ×10 magnification.

### 2.8 TG and Protein Content Evaluation

To measure the TG accumulation, cells were harvested by adding 200 μL/well of lysis buffer (0.01 M Tris–HCl pH 7.4, 0.15 M NaCl, 1 mM EDTA) containing protease inhibitors and disrupted by ultrasound for 5 s continuously at 40% of the instrument amplitude (Branson Digital Sonifier SFX 550). TG content was measured spectrophotometrically by using a commercial kit (Spinreact, Girona, Spain), whereas protein concentration was determined by Bradford assay [34]. Results are expressed as the percentage of the ratio between mg of TG and mg of total proteins, with respect to control cells, that is, cells differentiated in the absence of carotenoid extract.

### 2.9 Western Blot Analyses

For Western Blot analyses, at the end of the experiment, cells were harvested by adding 150 μL/well of RIPA buffer supplemented with protease and phosphatase inhibitors. After 30 min incubation on ice, vortexing every 5 min, samples were centrifuged at 14,000*g* for 15 min at 4°C. Samples were collected and protein concentration was determined by the BCA assay (Thermo‐Fisher, Waltham, MA, USA) [35]. Twenty micrograms of each sample were analyzed by SDS‐PAGE and electroblotted onto PVDF membranes (Millipore, Bradford, MA, USA). After blocking with 5% BSA, the following specific antibodies were used: anti‐CCAAT enhancer binding protein beta (c/EBPβ), anti‐peroxisome proliferator‐activated receptor gamma (PPARγ), anti‐adiponectin, anti‐phospho‐acetyl‐CoA carboxylase (pACC), anti‐acetyl‐CoA carboxylase (ACC), anti‐phospho‐hormone sensitive lipase (pHSL), anti‐hormone sensitive lipase (HSL), anti‐fatty acid synthase (FAS), and anti‐adipose triglyceride lipase (ATGL). Anti‐β‐Actin was used as loading control [36]. All antibodies were from Cell Signaling (Danvers, MA, USA). The chemiluminescence detection system was from Thermo Fisher Scientific (Rockford, IL, USA). Densitometric analyses were performed using a ChemiDoc system (Biorad Hercules, CA, USA).

### 2.10 RNA Extraction and RT‐PCR

Differentiated cells treated in the presence or absence of carotenoid extract were washed and harvested by adding 700 μL/well of TRIzol (Invitrogen, Carlsbad, CA, USA). The RNA was extracted, purified, and retrotranscripted as reported by González‐Arceo [37]. Briefly, RNA was extracted with TRIzol and quantified using an RNA 6000 Nano Assay (Thermo Scientific, Wilmington, DE, USA) and any possible DNA contamination was removed by a first step of DNase performed by a commercial kit (Applied Biosystems, Foster City, CA, USA). Then, 1.5 μg of total RNA was retrotranscribed by using the iScript cDNA Synthesis Kit (Bio‐Rad, Hercules, CA, USA). Relative *C/ebpβ*, *Srebf1*, *Pparγ*, *Adiponectin*, *Glut4*, *Acc*, *Hsl*, *Fas*, and *Atgl* mRNA levels were measured by Real‐Time PCR (RT‐PCR) (CFX96 Real‐Time System) at 60°C. All mRNA levels were normalized to β‐actin. The PCR mixture consisted of 4.75 μL of each cDNA, 5 mM of either the forward or reversed primer, and SYBR Green Master Mix (Applied Biosystems). Results are expressed as fold changes in threshold cycle (*C*_t_) values compared to the controls using the 2^−∆∆*C*t^ method [38]. Specific commercially synthesized primer sequences are reported in Table S1.

### 2.11 Statistical Analyses

Results are reported as the mean of three independent experiments (mean ± SD) and compared through one‐way analysis of variance (ANOVA) according to Bonferroni's method (post hoc) using GraphPad Prism for Windows, version 6.01. For cell viability, TG‐lowering effects, gene and protein expressions, each concentration tested is compared to control cells. No comparison among cell groups was carried out. Statistical significance was set at *p* ≤ 0.05.

## 3 Results

### 3.1 Carotenoids Extraction

*G. phlegrea* freeze‐dried biomass after proteins extraction (residual biomass) was used as starting material to recover carotenoids by ultrasound assisted extraction using ethanol (herein named CRE, Carotenoids Residual Extract). The same experiment was carried out in parallel on the fresh freeze‐dried biomass (herein named CFE, carotenoids fresh extract). Carotenoid extraction yields are reported in Table 1. Extraction yields were found to be very similar between the two biomasses, with a slight reduction in the case of the extraction performed on the residual biomass (18% ± 3% fresh biomass and 15% ± 3% residual biomass). Then, total carotenoid content in each extract was measured spectrophotometrically and reported as the ratio between mg of carotenoids and mg of extract. Results are reported in Table 1. It is interesting to notice that, even if the extraction yields were similar, a 40% increase in the total carotenoids content was observed in CRE with respect to CFE. This result is in line with our previous paper, in which, using a different solvent, a 40% increase was observed in the carotenoid content in the residual biomass with respect to the fresh one [27]. Then, the antioxidant activity of both extracts was analyzed by the in vitro ABTS assay, and the IC_50_ values were measured. Consistently, CRE exerted a strong antioxidant activity, with an IC_50_ of 47 ± 0.8 μg/mL, whereas the IC_50_ value of CFE was higher than 200 μg/mL. It is conceivable that the lower IC_50_ value may be related to zeaxanthin and β‐carotene content, previously quantified by HPLC, which is about 10 times higher in CRE with respect to the fresh one [27].

**TABLE 1:** Extraction yields, total carotenoids content, and IC_50_ of extracts obtained from *Galdieria phlegrea* fresh (CFE) and residual (CRE) biomass.

	Extraction yield (mg/mg_biomass_)	Carotenoids content (mg/mg_extract_)	IC_50_ (μg/mL)
Fresh biomass	18 ± 3	184 ± 22	219 ± 44
Residual biomass	15 ± 3	221 ± 17	47 ± 1

*Note:* Data shown are means ± SD of three independent experiments.

### 3.2 Tyrosinase Inhibitory Activity

The possible activity of *G. phlegrea* carotenoid extract in inhibiting tyrosinase activity was evaluated by an in vitro assay. Results clearly show that CRE was able to inhibit tyrosinase activity (IC_50_ 128 ± 25 μg/mL), whereas no inhibitory activity was observed with CFE, as no IC_50_ was calculated (> 300 μg/mL). On the other hand, the commercial inhibitor kojic acid was more efficient, with an IC_50_ value of 6.5 ± 0.3 μg/mL.

### 3.3 Carotenoid Extracts Biocompatibility

**FIGURE 1:**
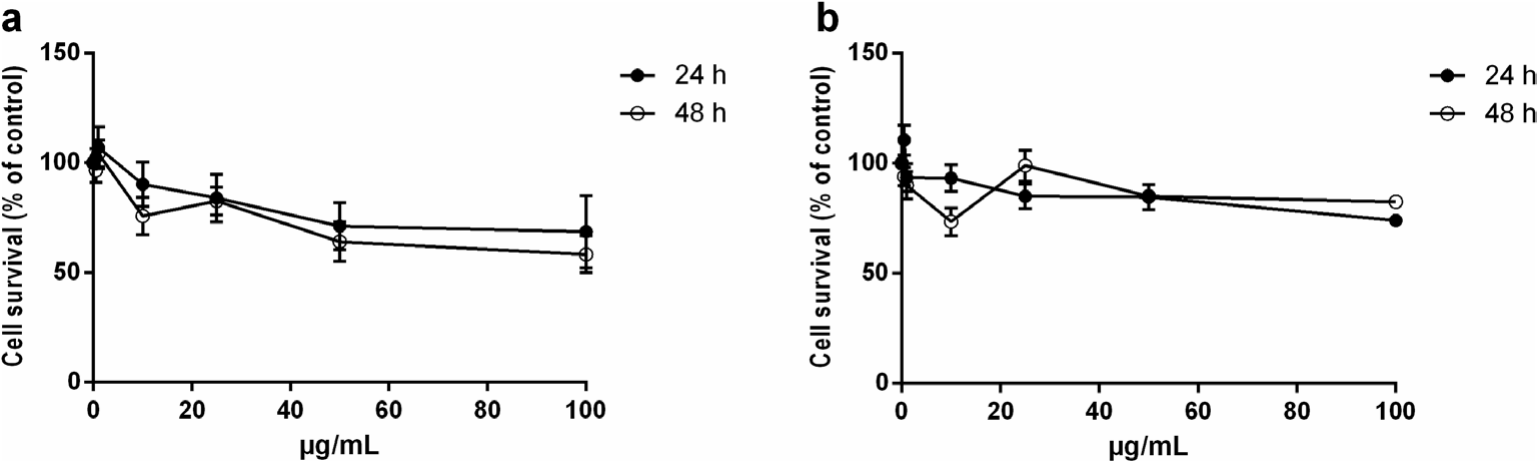
Effects of carotenoid extracts from *Galdieria phlegrea* on 3T3‐L1 cell viability. Dose–response and time‐dependent curves of 3T3‐L1 after 24 h (black circles) and 48 h (empty circles) incubation with increasing concentration of carotenoid extracts obtained from fresh (a) and residual (b) biomass. Cell viability was assessed by the MTT assay and cell survival was expressed as percentage of viable cells in the presence of ethanol extracts, with respect to control cells grown in the absence of the extract.

Carotenoid extracts, obtained from either fresh or residual *G. phlegrea* biomass, were tested on 3T3‐L1 preadipocytes, a cell‐based system widely used to study lipid accumulation. The evaluation of the biocompatibility of the carotenoid extracts on the proposed cell‐based model is a mandatory step needed to evaluate the concentration of the extracts to be used in the further experiments. To this purpose, cells were incubated with increasing concentrations of carotenoids (0.5–100 μg/mL) for 24 and 48 h. Cell viability was measured by the MTT assay, as described in Section 2. As reported in Figure 1a, CFE exerted a slight toxicity at high concentrations, whereas carotenoids extracted from the residual biomass were found to be fully biocompatible at any experimental condition analyzed (Figure 1b).

### 3.4 Effects of Carotenoids on 3T3‐L1 Preadipocyte Differentiation

**FIGURE 2:**
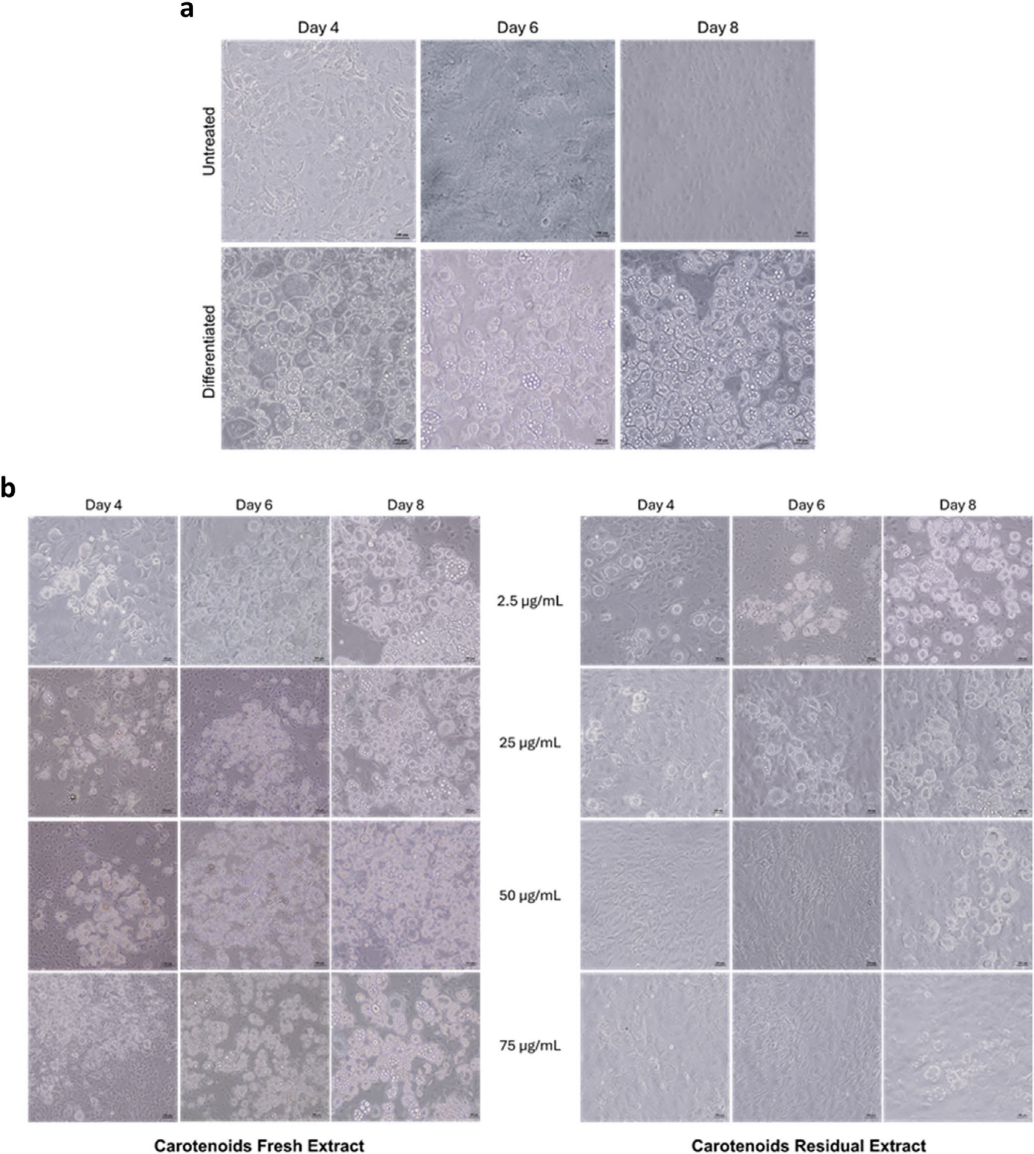
Optical microscopy images of 3T3‐L1 during the differentiation process. Effect of carotenoids extracted from *Galdieria phlegrea* fresh and residual biomass on 3 T3‐L1 cells differentiation process. Cells were differentiated in the absence (a) or presence (b) of 2.5, 25, 50, and 75 μg/mL of CFE (left panel) or CRE (right panel) for 8 days after confluence. Images were acquired by optical microscopy at ×10 magnification at Days 4, 6, and 8.

**FIGURE 3:**
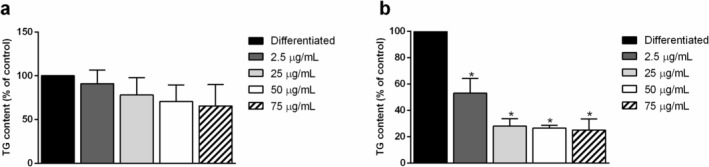
3T3‐L1 pre‐adipocyte triglyceride (TG) content after differentiation. TG accumulation was measured in cells after 8 days of differentiation, in the presence or absence of CFE (a) and CRE (b). Dark gray bars indicate cells incubated with 2.5 μg/mL of carotenoids extract, light gray bars cells incubated with 25 μg/mL of carotenoids extract, white bars cells incubated with 50 μg/mL and white dashed bars those with 75 μg/mL. Data are reported as the percentage of TG content with respect to cells differentiated in the absence of carotenoids (black bars), normalized with respect to the total protein content. Results are reported as means ± SD of three independent experiments. *p < 0.05 with respect to control cells.

The differentiation process of preadipocytes into mature adipocytes is a reversible and dynamic process in which, due to external triggers, cells store fatty acids in lipid droplets as energy storage [3]. As oxidative stress is considered the major cause of aging and is involved in most deleterious pathologies linked to lipid metabolism imbalance [39], carotenoids extracted from *G. phlegrea* were tested for their ability to affect preadipocyte differentiation. Due to changes in the extracellular and intracellular matrix, the occurrence of preadipocyte differentiation is clearly visible from the change in their morphology, as they go from a fusiform shape to a bigger and rounded one. Indeed, during the differentiation process, its progression was monitored, and images were acquired by optical microscopy (×10 magnification) at Days 4, 6, and 8. As shown in Figure 2a, when cells were differentiated, lipid accumulation was observed starting from Day 4. When cells were differentiated in the presence of increasing CFE concentrations (2.5–75 μg/mL) the formation of lipid droplets seemed to be slower, as a lower amount of lipid droplets was observed at Day 4, independently from the concentration of carotenoid extract tested, with respect to cells differentiated in the absence of any treatment. However, even if at a slower pace, an almost complete differentiation was observed at Day 8. Intriguingly, when cells were differentiated in the presence of CRE, the process was almost inhibited, as no lipid droplets were observed up to Day 6 at any concentration tested. At Day 8, a very low amount of lipid droplets was observed in cells incubated with up to 25 μg/mL carotenoid extract, and almost no droplets were observed at 50 and 75 μg/mL (Figure 2b). At the end of differentiation, total TG content was measured. As shown in Figure 3, TG content is reported as the percentage of the ratio between mg of TG and mg of total proteins with respect to the ratio obtained for control cells (i.e., cells differentiated in the absence of carotenoid extract). According to the phenotype observed, no significant inhibition in the TG content was measured when cells were differentiated in the presence of CFE at any concentration tested (Figure 3a). Remarkably, a significant inhibition in the total TG content was measured in cells differentiated with carotenoid extract obtained from the residual biomass, starting from 2.5 μg/mL (about 50%), but at 25 μg/mL the inhibition reached a plateau (about 70%). Thus, this concentration was chosen for further analyses as this represents the lowest active concentration in the case of CRE.

### 3.5 Effect of Carotenoids on the Expression of Proteins Related to Adipogenesis

**FIGURE 4:**
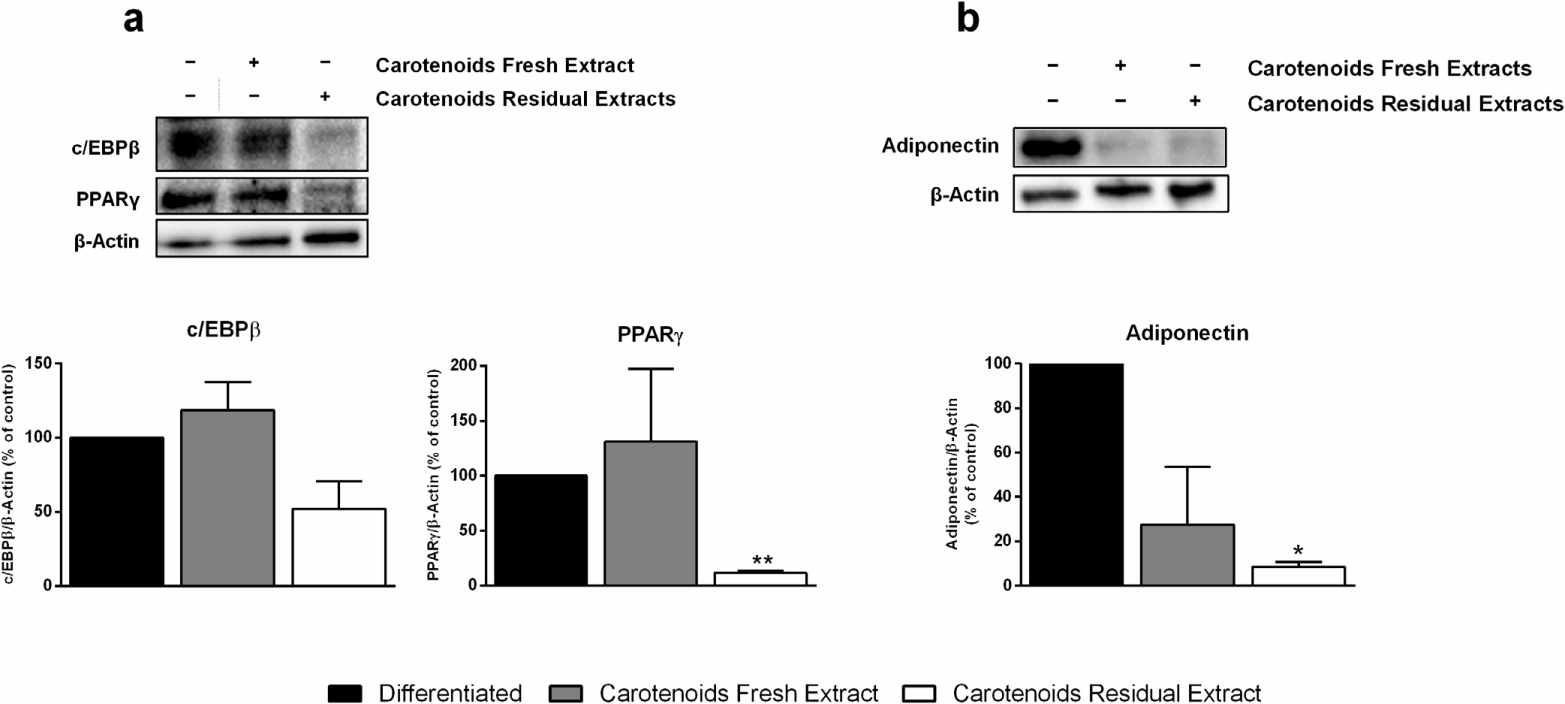
Effect of carotenoids on c/EBPβ, PPARγ and adiponectin activation in 3T3‐L1 cells. Cells were differentiated as reported in Section 2 and incubated with 25 μg/mL of CFE (gray bars) and CRE (white bars) during all the differentiation steps. Cells were analyzed by immunoblotting with specific antibodies against (a) c/EBPβ and PPARγ and (b) adiponectin. β‐actin was used as an internal standard. The relative densitometric analysis of c/EBPβ, PPARγ, and adiponectin is reported. Black bars refer to cells differentiated in the absence of any extract and used as control. Data shown are the means ± SD of three independent experiments. *p < 0.05, **p < 0.005 with respect to control cells.

Cells were incubated in the presence or absence of 25 μg/mL of CFE or CRE of *G. phlegrea*. As shown in Figure 4, carotenoids extracted from fresh and residual biomass exert different effects on the protein expression of two transcription factors involved in adipogenesis. In particular, the treatment with CFE did not modify the expression of c/EBPβ and PPARγ when compared to the control cells. By contrast, when cells were differentiated in the presence of the same concentration of CRE, a reduction of about 50% and 80% of c/EBPβ and PPARγ levels, respectively, was observed (Figure 4a). Conversely, adiponectin expression was reduced upon treatment with both carotenoid extracts (Figure 4b), although statistical significance was only found in the case of CRE (about 90%).

### 3.6 Effects of Carotenoids on the Expression of Genes Related to Adipogenesis in 3T3‐L1 Preadipocytes

**FIGURE 5:**
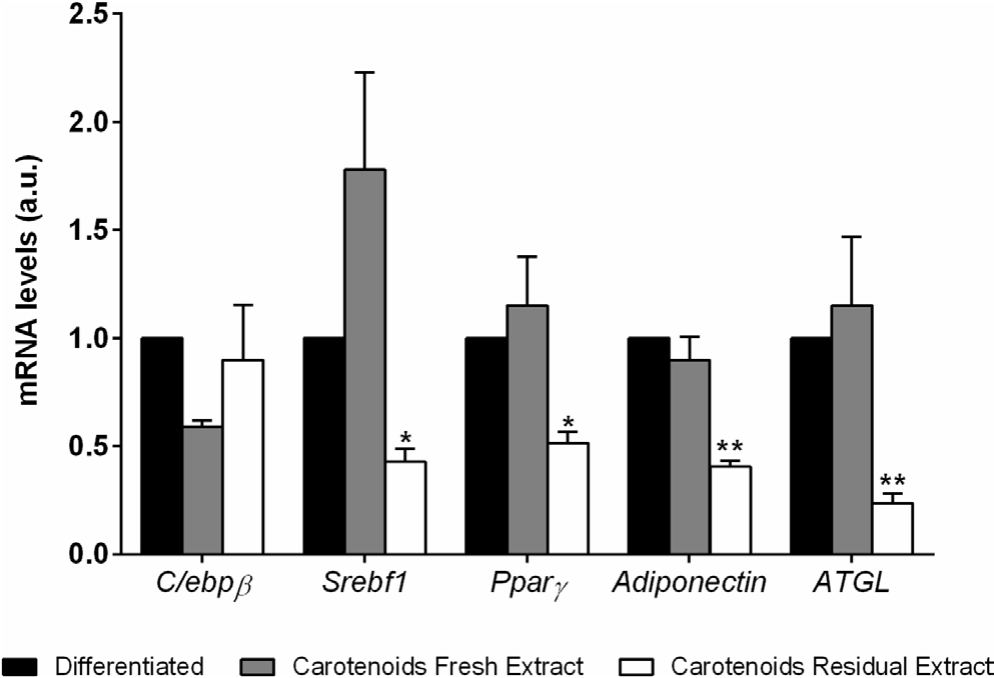
Effects of *Galdieria phlegrea* carotenoids extract on gene expression in pre‐adipocytes. Relative mRNA levels of *C/ebpβ*, *Srebf‐1*, *Pparγ*, *Adiponectin*, and *Atgl* in maturing 3T3‐L1 differentiated for 8 days in the presence or absence of 25 μg/mL of carotenoids extracted from fresh (gray bars) and residual (white bars) biomass of *G. phlegrea*. Results are expressed as fold changes in threshold cycle (*C*_t_) values compared to the controls (black bars) using the 2^−∆∆*C*t^ method. Data shown are means ± SD of three independent experiments. *p < 0.05, **p < 0.005 with respect to control cells.

To better investigate if carotenoid extracts could exert their activity at transcriptional levels, mRNA levels of genes involved in the adipogenic pathway were measured by RT‐PCRs. Genes involved in the early (*C/ebpβ*, *Srebf‐1*) and medium (*Pparγ*) adipogenesis transcriptional cascade were analyzed, as well as *Adiponectin* and *Atgl*, involved in the maturation process. Results are reported in Figure 5. When cells were differentiated in the presence of CFE, no changes were observed in the expression of the analyzed genes. By contrast, when cells were treated with CRE, a significant decrease in the mRNA levels of all the analyzed genes was observed, apart from c/EBPβ.

### 3.7 Effects of Carotenoids on Mature Adipocytes

**FIGURE 6:**
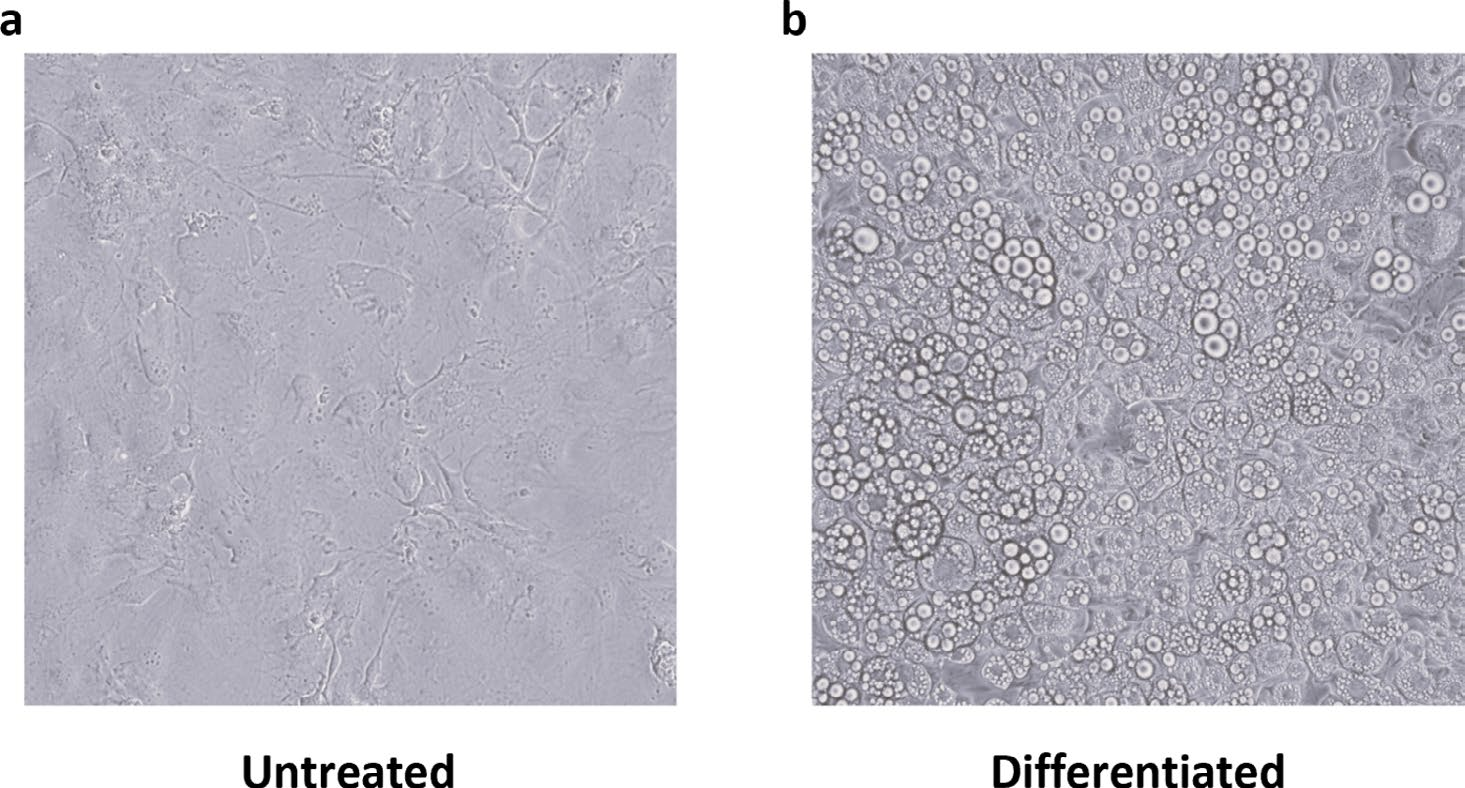
Optical microscopy images of 3T3‐L1 differentiated cells. (a) Cells grown in DMEM; (b) cells grown in differentiation medium after 12 days. Images were acquired by optical microscopy at ×10 magnification.

**FIGURE 7:**
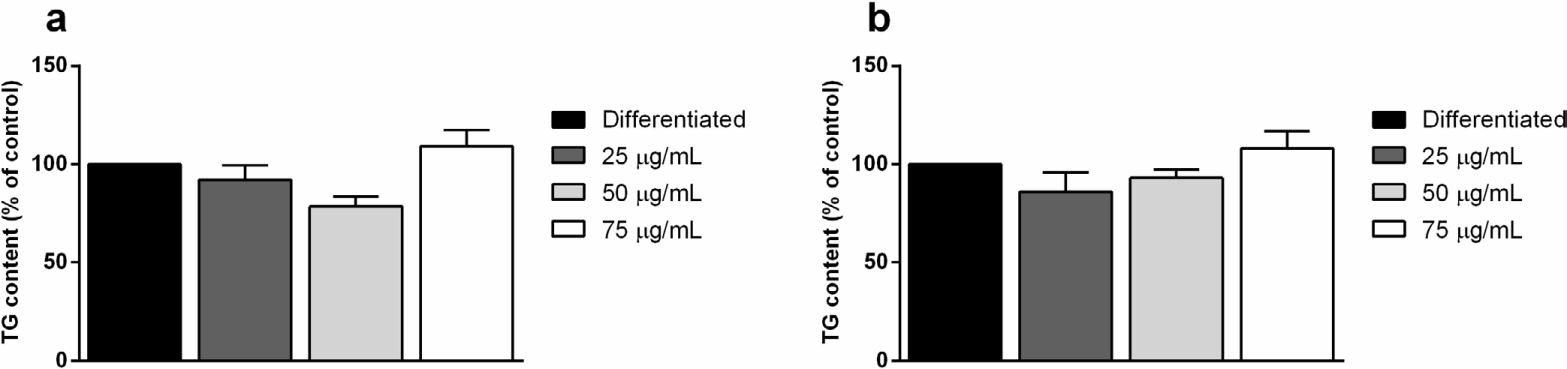
Effects of carotenoids from *Galdieria phlegrea* on 3T3‐L1 mature adipocytes triglyceride content. Triglyceride accumulation on mature adipocytes after 24 h of treatment with 25 μg/mL (dark gray bars), 50 μg/mL (light gray bars) or 75 μg/mL (white bars) of carotenoids extracted from *G. phlegrea* biomass. (a) CFE, (b) CRE. Data are reported as the percentage of TG content with respect to cells differentiated in the absence of carotenoids (black bars). Results are reported as means ± SD of three independent experiments.

Besides the effects of the extracts on preadipocyte maturation, their effect on mature adipocytes was evaluated. To this purpose, cells were differentiated as reported in the Section 2 until Day 12, and then treated with 25, 50, and 75 μg/mL of CFE or CRE for a further 24 h. Cells differentiated in the absence of carotenoids (Figure 6b) showed a visible change in their morphology with the formation of a higher number of lipid droplets, indicating the differentiation into mature adipocytes. After 24 h treatment with carotenoids, TG content was measured, but the results showed no significant differences in total TG content among the analyzed samples (Figure 7).

### 3.8 Effects of Carotenoids on Proteins Involved in TG Metabolism

**FIGURE 8:**
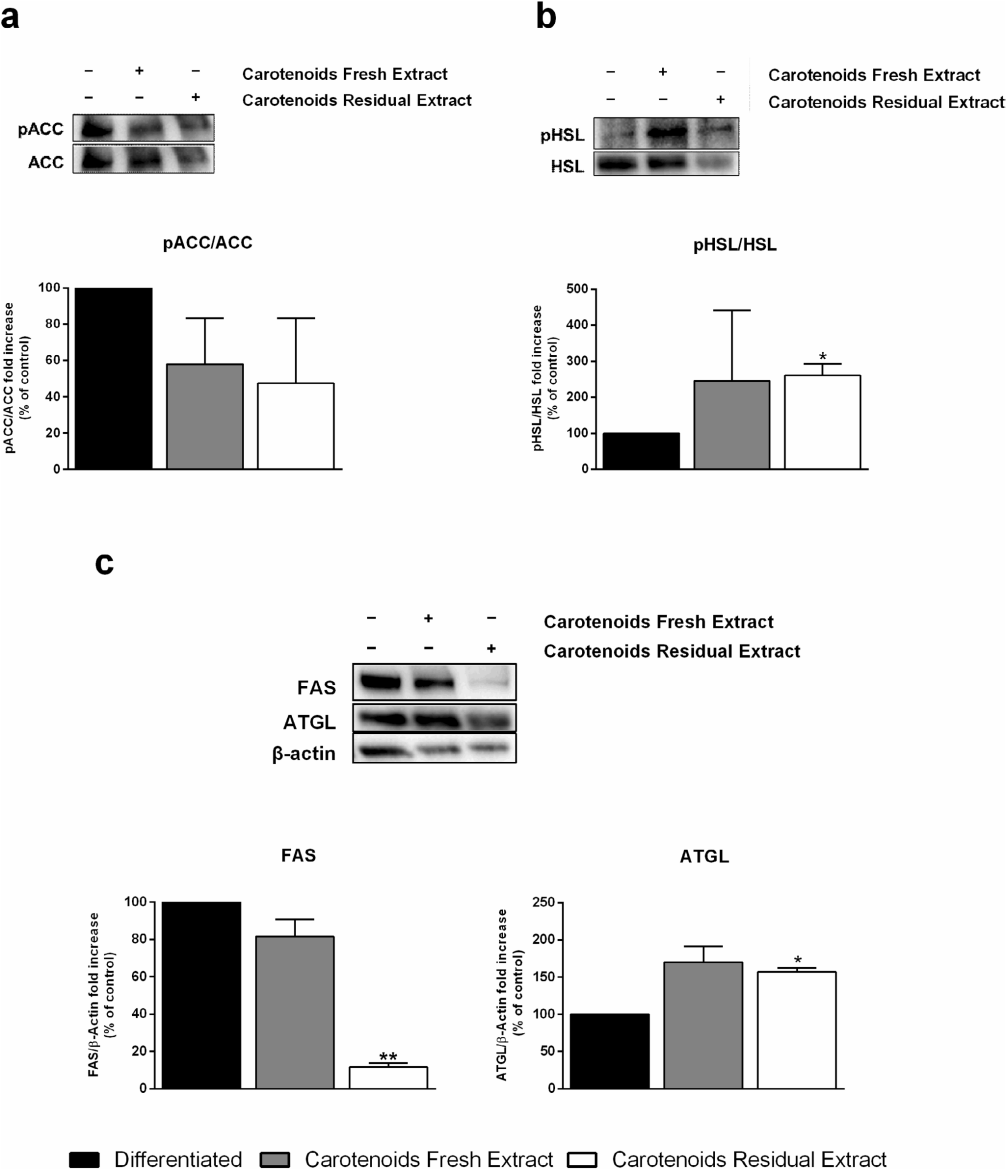
Effect of carotenoids on 3T3‐L1 mature adipocytes. 3T3‐L1 mature adipocyte were incubated for 24 h with 25 μg/mL of carotenoids extracted from fresh (gray bars) and residual (white bars) *Galdieria phlegrea* biomass. Western blots show the phosphorylation levels of ACC (a) and HSL (b) with respect to their total amount; FAS and ATGL (c) are normalized with respect to β‐actin (used as internal standard). The relative densitometric analysis is reported for differentiated cells (black bars), cells treated with CFE (gray bars), and CRE (white bars). Phosphorylated proteins are normalized with respect to their total form. Data shown are the means ± SD of three independent experiments. *p < 0.05 and **p < 0.005.

In order to investigate the de novo lipogenesis, two major proteins were analyzed, FAS, which exerts its activity in the first steps of fatty acid synthesis, and ACC, the limiting enzyme of the process. ACC is inactivated upon phosphorylation; thus, the changes in the pACC/ACC ratio were measured as an indicator of its activity. As reported in Figure 8a, no significant difference in the pACC/ACC ratio was observed for both extracts with respect to differentiated cells, whereas FAS expression, reported in Figure 8c, significantly decreased in cells treated with CRE compared to those obtained from the fresh biomass (about 90% decrease). The lipolytic pathway was analyzed by verifying changes in the levels of the two major lipases, adipose trygliceride lipase (ATGL) and hormone‐sensitive lipase (HSL). As HSL is activated by phosphorylation, the ratio pHSL/HSL was used as an indicator of its activity. This ratio was significantly increased after treatment with CRE but not with CFE (Figure 8b). As shown in Figure 8c, CRE significantly increased ATGL expression, whereas no significant increase was observed in cells treated with the extract from the fresh biomass.

### 3.9 Effects of Carotenoids on Lipid Metabolism Genes

**FIGURE 9:**
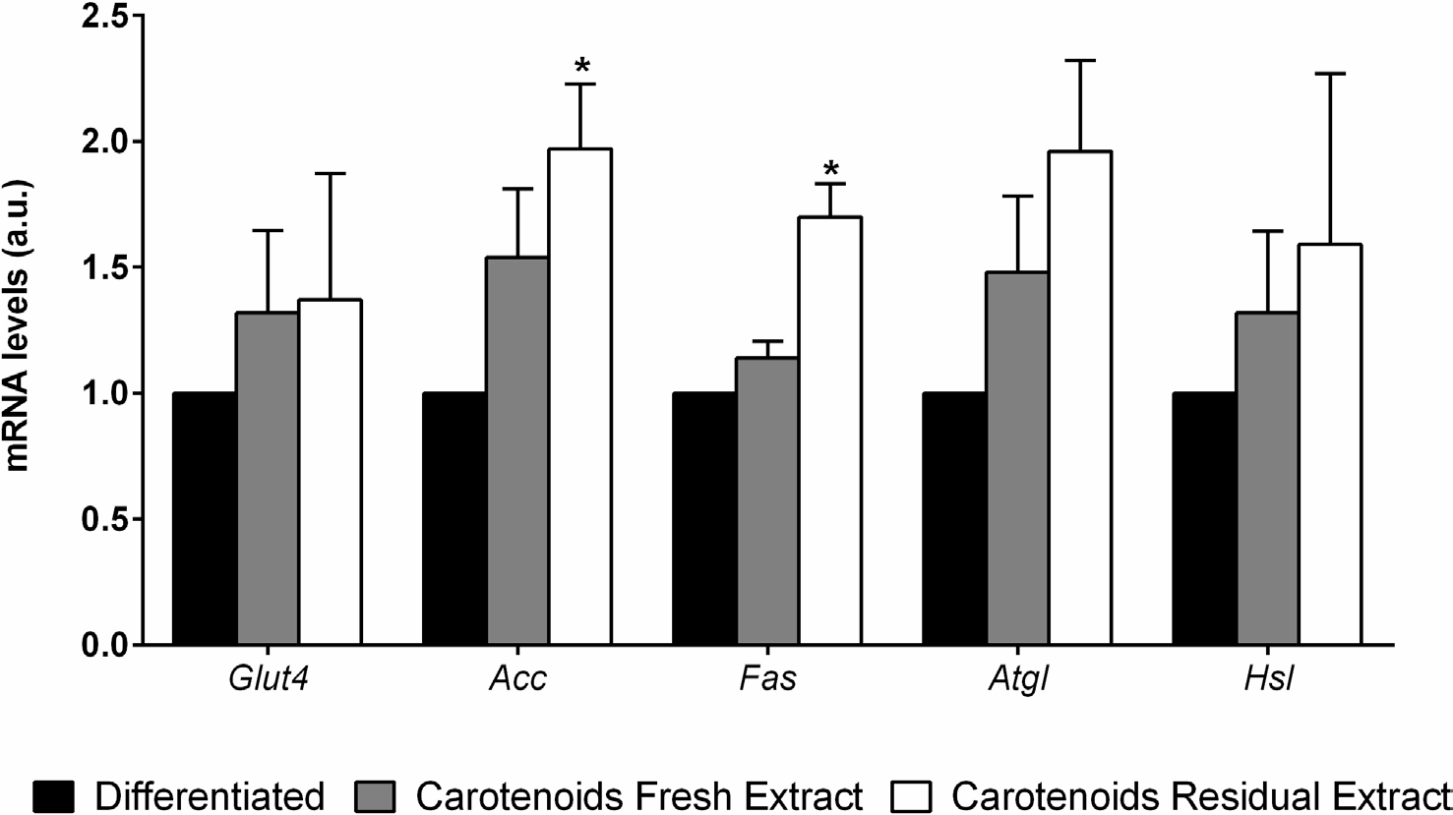
Effects of *Galdieria phlegrea* carotenoids on lipid metabolism genes. Relative mRNA levels of *Glut4*, *Acc*, *Fas*, *Atgl*, and *Hsl* in mature 3T3‐L1 after 24 h treatment with 25 μg/mL of carotenoids extracted from the fresh (gray bars) and residual (white bars) biomass of *G. phlegrea*. Results are expressed as fold changes in threshold cycle (*C*_t_) values compared to the controls (black bars) using the 2^−∆∆*C*t^ method. Data shown are the means ± SD of three independent experiments. *p < 0.05.

Finally, the expression of genes involved in de novo lipogenesis and lipolysis, as well as that of glucose transporter type 4 (Glut4), involved in the glucose uptake, was measured. As shown in Figure 9, *Acc* and *Fas* were significantly upregulated in mature adipocytes treated with the CRE extract.

## 4 Discussion

In the cosmeceutical industry, pigments are widely used because of their antioxidant, anti‐inflammatory, anti‐proliferative, and antimicrobial activities [40, 41] and are mainly obtained from nonrenewable synthetic sources [42] for their low production costs. However, the increase in their demand to cover industrial production has boosted concerns about environmental impact and safety issues for human health due to their toxicity [43]. For this reason, the transition toward the use of natural pigments is overriding to cover consumers' demand. As already mentioned, microalgae are a great source of high‐value products with a variety of applications. In this work, we focused our attention on carotenoids extracted from *G. phlegrea* [27]. In a previous paper, the carotenoids present in CFE and CRE were identified and quantified by HPLC and mass spectrometry analysis [27]. The extraction yield was similar in both extracts, which were found to be enriched in β‐carotene and zeaxanthin. Surprisingly, the extraction on the residual biomass allowed increasing more than 10 times the amount of β‐carotene and zeaxanthin with respect to that obtained from the fresh biomass [27]. As the previously reported extract was endowed with a strong antioxidant activity, an in vitro antioxidant assay was done on both extracts. The ABTS assay clearly indicates that CRE is endowed with a stronger antioxidant activity than CFE. Oxidative stress can be considered the starting point of different health conditions, such as skin disorders [44]. For this reason, carotenoids from *G. phlegrea* were tested in vitro as inhibitors of tyrosinase, a well‐known enzyme involved in skin hyperpigmentation and melanin formation via redox reactions [45]. Although melanin is an essential molecule for humans, alteration in melanogenesis can cause different dermatological problems, such as lentigo, hyperpigmentation due to skin aging, and, in the worst cases, also melanoma [46]. Therefore, tyrosinase inhibitors are considered potential skin‐whitening agents, largely used in the cosmetic industries [47]. When tested in vitro, CRE was able to inhibit tyrosinase, even though to a lower extent than Kojic acid, used as a control. Interestingly, CFE did not exert any inhibitor effect.

Another common skin disorder, linked to oxidative stress, is GLD, which affects more than 80% of postpuberal females [48]. GLD, in turn, is mainly related to adipogenesis and lipid metabolism alteration, regulated at a very sophisticated level. 3T3‐L1 cells represent a valuable model system to study preadipocyte differentiation as their biochemical pathway is well defined. Thus, cell differentiation was studied in the presence of *G. phlegrea* carotenoid extracts. Our results pointed out that the incubation of pre‐adipocytes with CRE showed a strong activity in counteracting adipocyte differentiation in the 3T3‐L1 adipocyte model system and demonstrated the molecular mechanisms underlying the inhibition of differentiation. CRE was able to significantly inhibit TG accumulation during pre‐adipocyte differentiation acting on the gene and protein expression of key transcriptional factors involved in the pathway [49]. Among them, CCAAT/enhancer‐binding protein β (c/EBPβ) plays a pivotal role in inducing cell differentiation as it is the first protein activated after differentiation stimuli. c/EBPβ can activate the expression of peroxisome proliferator‐activated receptors (PPARs), with PPARγ as the master regulator of adipogenesis [50]. Another key protein in the adipogenesis pathway is the sterol regulatory element‐binding protein 1 (SREBP1), also called adipocyte determination and differentiation‐dependent factor 1 (ADD1), that is predominantly expressed in adipose tissues with a key role in regulating the expression of lipid metabolism proteins (FAS and LPL) and prompting adipocyte differentiation through a specific upregulation of PPARγ expression [51]. In the presence of external ligands (e.g., intracellular free fatty acids, metabolites, antidiabetic drugs) PPARγ changes its tridimensional conformation and translocates to the nucleus where it binds to PPAR response element sequences, thus activating the transcription of adipocyte differentiation genes (e.g., adiponectin and PPARγ itself) [52]. The analysis of adipogenic transcriptional factors reveals that CRE can interfere with the differentiation process, as *Pparγ* and *Srebf‐1* gene expressions were significantly decreased, as well as adiponectin and ATGL, genes related to adipocyte maturation. These results clearly indicate that CRE is able to regulate the process at a transcriptional level, even if a translational regulation cannot be excluded. We may hypothesize that the antiadipogenic activity in CRE is related to the presence in the extract of zeaxanthin and β‐carotene, as, in a previous paper, we clearly demonstrated that the two synthetic carotenoids were effective in counteracting oxidative stress only when combined together [27]. Our results are in line with those obtained by using synthetic carotenoids. As an example, Liu revealed the efficacy of zeaxanthin on the same experimental model [53], whereas Gopal and colleagues tested synthetic β‐carotene and lutein (zeaxanthin isomer), with lutein being the only active molecule [54].

When CRE was evaluated on mature adipocytes, no TG reduction was observed. Longer incubations (48 and 72 h) were also tested, but no changes in TG content were observed (data not shown). In the protocol used, the differentiation process lasts 12 days, during which a significant increase in TGs was observed. Upon a 24 h treatment with CRE, the synthesis of two major lipases (HSL in its active form and ATGL) is activated, and FAS synthesis is inhibited. However, upon 24 h incubation, it is conceivable that no TG reduction is observed, as phenotype changes require more time and may not be strictly linked to a lack of activity. Thus, changes in gene and proteins involved in the de novo lipogenesis (*Acc, Fas*) were analyzed in mature adipocytes upon incubation with CRE. Results indicated that the mRNAs related to both enzymes were upregulated. Intriguingly, the ratio pACC/ACC did not change upon incubation with CRE, whereas a significant decrease in FAS protein expression was observed. Thus, it can be proposed that CRE is able to reduce the last step of the *de novo* lipogenesis at a translational level.

When the lipolytic pathway was analyzed, although no changes in *Atgl* and *Hsl* gene expression were observed, ATGL protein expression increased, along with an increase in pHSL/HSL, suggesting the activation of the pathway. The decrease in de novo lipogenesis and the increase in lipolysis, in contrast with the nonalteration of TG content, allow us to hypothesize that a phenotypical change may require a longer incubation time to be visible.

Different results were obtained with CFE. During the differentiation process, CFE showed a TG accumulation slower than that observed in control cells, as a lower amount of lipid droplets was observed at Day 4. In spite of that, a complete differentiation was observed at Day 8, suggesting that CFE did not show an antiadipogenic effect. Accordingly, at the end of the differentiation process, no change in genes and proteins related to differentiation was observed. The effect of CFE was tested also on mature adipocytes, and no effects were observed on TG accumulation, protein, or gene expression.

To date, few papers report the antiobesity activity of microalgae extracts obtained by a single extraction. Sugimoto and colleagues reported that a 20% aqueous extract from the green microalga *Euglena gracilis* strongly inhibited the early stage of adipocyte differentiation in human adipose‐derived stem cells [55]. The organic fraction extracted from the green microalga *Haematococcus pluvialis* (20 μg/mL) was found to be effective on both 3T3‐L1 pre‐adipocyte differentiation and on differentiated adipocytes, decreasing lipogenesis and increasing lipolysis [56]. An ethanol extract from the cyanobacterium *Spirulina maxima* was able to inhibit 3T3‐L1 cell differentiation, even if a higher concentration of the extract was used (up to 100 μg/mL) [57]. Noteworthy, the extract used by Seo and colleagues has a different chemical composition, as it contains C‐Phycocyanin and chlorophyll *a*, whereas CRE contains β‐carotene and zeaxanthin. It should be pointed out that, despite the well‐recognized healthy effects of microalgae compounds, microalgae require high cultivation costs and contain low concentrations of bioactive molecules, so that the extraction of a single class of molecules may not be remunerative and not feasible at an industrial scale. To the best of our knowledge, for the first time, carotenoids obtained in a cascade approach from the residual biomass of *G. phlegrea* were used on the 3T3‐L1 cell model and found to be effective in counteracting adipogenesis, de novo lipogenesis, and enhancing lipolysis, as well as interfering in vitro against skin hyperpigmentation.

In conclusion, this work stresses the importance of a biorefinery approach not only to debottleneck microalgae limitations, but also to obtain extracts able to counteract adipogenesis and suggests CRE as an excellent candidate toward potential new products for cosmeceutical formulations.

## Author Contributions

**Enrica Giustino:** writing original draft, validation, methodology, investigation, formal analysis and data curation. **Paola Imbimbo:** writing review and editing, supervision, investigation, formal analysis, and conceptualization. **Jenifer Trepiana:** writing review and editing and supervision. **Maria Puy Portillo:** writing review and editing, supervision, conceptualization, and resources. **Daria Maria Monti:** writing review and editing, supervision, project administration, resources, and conceptualization. All authors read and approved the manuscript.

## Conflicts of Interest

The authors declare no conflicts of interest.

## Supporting information


**Data S1.**Supporting Infromation.

## Data Availability

All data supporting the findings of this study are available within the paper and in the Supporting Information. Additional details can be provided by the corresponding authors upon reasonable request.
