# Supplementary material for: Unlocking the Antiadipogenic Potential of Carotenoids From Galdieria phlegrea
Source: Biofactors. 2025 Jun 5;51(3):e70027. doi: 10.1002/biof.70027 (PMC12138963; doi:10.1002/biof.70027)
Supplement: Supplementary file 1 — Data S1.Supporting Infromation. [file BIOF-51-0-s001.pdf]

Supplementary Information

Unlocking the anti-adipogenic potential of carotenoids from *Galdieria phlegrea*

Enrica Giustino[1], Paola Imbimbo[1], Jenifer Trepiana[2,3,4], Maria Puy Portillo[2,3,4*], Daria Maria Monti[1*]

[1] Department of Chemical Sciences, University of Naples Federico II, via Cinthia 4, 80126, Naples, Italy.

[2] Nutrition and Obesity Group, Department of Nutrition and Food Science, Faculty of Pharmacy and Lucio Lascaray Research Center, University of the Basque Country (UPV/EHU), 01006 Vitoria-Gasteiz, Spain.

[3] CIBERobn Physiopathology of Obesity and Nutrition, Institute of Health Carlos III, 28222 Madrid, Spain

[4] BIOARABA Health Research Institute, 01006 Vitoria-Gasteiz, Spain

*Corresponding authors: Maria Puy Portillo, mariapuy.portillo@ehu.eus; Daria Maria Monti, mdmonti@unina.it

Table S1 Primer sequences for real-time PCR genes

Gene	Sense primer	Antisense primer
C/ebpβ	5'-CAAGCTGAGCGAGTCCA-3'	5'-CAGCTGCTCCACCTTCTTCT-3'
Srebf-1	5'-GTCGTTGGCATCCTGCTATC-3'	5'-TAGCTGGAAGTGACGGTGGT-3'
Pparγ	5'-TCGCTGATGCACTGCCTATG-3'	5'-GAGAGGTCCACAGAGCTG-3'
Adiponectin	5'-GACGACACCAAAAGGGTCCA-3'	5'-GAGTGCCATCTCTGCCA-3'
Atgl	5'-GAGCTTCGCGTCACCAAC-3'	5'-CACATCTCTCGGAGGACC-3'
Glut4	5'-GTCCTCACAGTACTCCCTGC-3'	5'-AGGTATCTGGGGCTCTCAGG-3'
Acc	5'-GGAGCCAGAAGGGACAGTAG-3'	5'-CAGCCAAGCGGATGTAAACT-3'
Hsl	5'- AGCCCCTCAAGTGCACAGTG-3'	5'- TGCCAATGTGTTTTCCCTGA-3'
Fas	5'-AGCCCCTCAAGTGCACAGT-3'	5'-TGCCAATGTGTTTTCCCTG-3'
β-actin	5'-CCCGCGAGTAGAACCTTCT-3'	5'-CGTCATCCATGGCGAACT-3'

C/ebpβ: CCAAT Enhancher Binding Protein Beta; *Srebf-1*: Sterol Regulatory Element-Binding Protein 1; *Pparγ*: Peroxisome Proliferator-Activated Receptor gamma; *Atgl*: Adipose Trygliceride Lipase; *Glut4*: Glucose Transporter Type 4; *Acc*: Acetyl-CoA Carboxylase; *Hsl*: Hormone Sensitive Lipase; *Fas*: Fatty Acid Synthase
